# Wild and weedy *Hesperis matronalis* hosts turnip mosaic virus across heterogeneous landscapes in upstate New York

**DOI:** 10.1016/j.virusres.2022.199011

**Published:** 2022-11-28

**Authors:** Elizabeth M. Lombardi, Jasmine Peters, Lukin Jacob, Alison G. Power

**Affiliations:** Cornell University, Department of Ecology and Evolutionary Biology, E145 Corson Hall, Ithaca, New York 14853, USA

**Keywords:** Turnip mosaic virus, *Hesperis matronalis,* Wild host plants, Tolerance, Spillover, Plant virus ecology, Virus spillover, Wild viruses

## Abstract

Turnip mosaic virus (TuMV) is a widespread and economically important pathogen in agricultural crops and has the widest known host range in the virus family *Potyviridae*. While management of the virus and its aphid vectors in agricultural fields decreases virus incidence, many alternative wild hosts for TuMV may serve as source populations for crop infection through spillover. Over thirty years ago, research demonstrated that the introduced brassica, Dame's Rocket (*Hesperis matronalis)* hosts several viruses, including TuMV. Here, we use both enzyme-linked immunosorbent assays (ELISA) and next generation sequencing to document the frequent infection by TuMV of Dame's Rocket, which is common and widespread in disturbed areas around crop fields in upstate New York. Deep sequencing of multiple tissue types of symptomatic hosts indicate that the infection is systemic and causes diagnostic, visible symptoms. In a common garden experiment using host populations from across upstate New York, we found evidence for genetic tolerance to TuMV infection in *H. matronalis*. Field surveys show that TuMV prevalence varies across populations, but is generally higher in agricultural areas. Examining disease dynamics in this and other common alternative hosts will enhance our understanding of TuMV epidemiology and, more broadly, virus distribution in wild plants.

## Highlights

• Dame's Rocket, *Hesperis matronalis*, is a widespread host of turnip mosaic virus.
• TuMV prevalence is higher in hosts growing in agricultural landscapes.
• Infection with TuMV induces leaf mottle and color breaking floral phenotypes.
• We present evidence for genetically-mediated tolerance of TuMV across hosts.

## 1 Introduction

Plant viruses are ubiquitous and can be consequential for both agricultural and wild host species (Roossinck, 2019, 2011). Generalist viruses that infect diverse host species are particularly concerning given the potential for i) range expansions into new hosts or habitats and ii) spillover between wild plants and crops. Until relatively recently, the prohibitive cost of *de novo* detection of viruses in wild hosts inhibited full documentation of the diversity and distribution of plant viruses across biogeographical space and between diverse host taxa (but see Roossinck, 2011 for an early review of the potential benefits of sequencing; and (Kutnjak et al., 2015; Pecman et al., 2017) for the first agricultural study using next generation sequencing (NGS). Though this clear gap in our ecological understanding of wild plant viruses has prompted calls for more research (Hasiów-Jaroszewska et al., 2021; Koonin and Dolja, 2018; Lefeuvre et al., 2019; Shates et al., 2019; Stobbe and Roossinck, 2014), data remain sparse. In particular, there is proportional underrepresentation of perennial dicots in wild plant virus research (Shates et al., 2019) despite high dicot abundance and species diversity in wild plant communities. We describe here a pathosystem that will contribute to filling this empirical gap, providing an example of a wild plant-virus interaction that occurs across a broad biogeographical range.

The biennial *Hesperis matronalis (*Brassicaceae*)*, commonly called Dame's Rocket, was introduced to North America in the late 17th century (Gleason and Cronquist, 1963) when European colonizers established gardens with seeds brought from Europe. Dame's Rocket produces a basal rosette during its first growing season, then multiple showy inflorescences with compound flowers that vary from white to deep purple during the second, and final, growing season. In addition to producing attractive flowers, *H. matronalis* is tolerant of a range of light, soil and water conditions, which made it a desirable garden species in settler gardens of the Allegheny Plateau and northern Appalachian mountain chain. Prolific and hardy, the plant soon escaped cultivated areas and has since undergone a range expansion across North America.

**Fig. 1:**
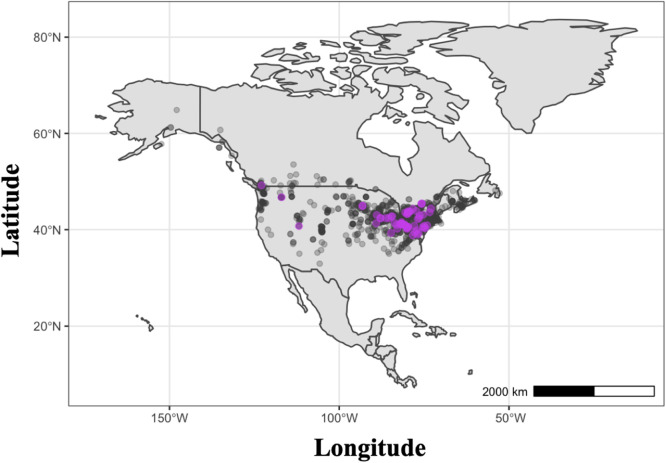
Distribution of *H. matronalis* populations across North America (dark grey dots) and locations where visual presence of color breaking flower petals were detected, indicating TuMV presence in *H. matronalis* individuals (purple dots). Host occurrence data were sourced from online biodiversity repositories.

Disturbed areas from Alaska to northern Mexico, and from the Atlantic to the Pacific coast, now harbor wild populations of this plant (Fig. 1).

In 1988, serological tests confirmed that *H. matronalis* can host multiple viruses, including the potyvirus turnip mosaic virus (TuMV) (Ford, 1988), though the virus had been isolated from agricultural hosts on the North American continent well before this (Schultz, 1921). Turnip mosaic virus is a single stranded RNA virus transmitted by a variety of insect vectors in a non-persistent manner, and is known to infect and cause disease symptomology in a wide range of host species (Nellist et al., 2022). In Ford's preliminary assessment of virus diversity in *H. matronalis,* it was hypothesized that flower color variegation may be either a symptom of a potyvirus association or a result of host genetic mutation. In the interim years, however, no further research has explored TuMV incidence or the impact of infection in *H. matronalis* despite host range expansions and persistent TuMV infections in crops across North America (Tomlinson and Walker, 1973; Walsh and Jenner, 2002). This absence of biogeographical research is not surprising given the challenge of studying viruses in wild hosts across broad spatial scales.

In this study, we survey *H. matronalis* across multiple landscape types using multiple virus detection tools to assess TuMV prevalence in the expanded range of *H. matronalis*. We also assess environmental versus genotypic effects on host response to TuMV through experimental infections of *H. matronalis*. We test the hypotheses that i) flower color breaking and leaf mottling are symptoms of TuMV in *H. matronalis* and ii) host genotype is a stronger driver of *H. matronalis* susceptibility to virus infection than host environment.

## 2 Materials and methods

### 2.1 Field surveys and initial sample collections

We carried out virus surveys in wild brassica populations in June and September 2016 and June 2018 at sites throughout Tompkins, Seneca, Hamilton, Essex and St. Lawrence counties in upstate New York. In 2016 we sampled eleven sites, but the relative size of host clusters in the Adirondack Mountain region (Hamilton, Essex and St. Lawrence counties) were small compared to those in the Finger Lakes (Seneca and Tompkins counties), and thus an altered sampling method was required in the survey in 2018 (see sampling scheme in Table S3).

In June 2016, where host groups of similar sizes (∼15 meter diameter with more than 100 individuals) could be located, ten replicate 1 meter by 1 meter quadrats were placed approximately 1 meter apart along a transect running from the center to the edge of the cluster. We collected leaf tissue from all plants belonging to family *Brassicaceae* in each quadrat. While the survey was primarily focused on assaying *H. matronalis*, we also included other confamilial hosts whenever they occurred within the quadrats. We tested 434 individual plants for viral infection including individuals of brassica species *H. matronalis, Alliaria petiolata, Sinapis arvensis, Brassica rapa* and *Capsella bursa-pastoris.* In subsequent surveys (late 2016 and all of 2018) we focused on *H. matornalis* only. Leaf tissue from every *H. matronalis* individual in the sparse Adirondack populations were included in the late 2016 survey (N=33 individual plants). To standardize sampling methods in 2018, equal numbers of *H. matronalis* plants (N=10 per population) were sampled along 10 meter transects placed randomly through host populations (Table S3).

In both surveys in 2016, we sampled leaf tissue from 50 individuals in each of six large populations in the Finger Lakes region and five populations of smaller size (5-20 individuals per population) in the Adirondack region. In 2018, we sampled ∼10 individuals from each of nine populations in the Finger Lakes region and four populations in the Adirondack region, plus full surveys of all volunteer *H. matronalis* within five meters of an experimental field (not included in the survey) in each region (N=243 total plants sampled).

From each host sampled, whole leaves were collected and stored in a cooler for up to 18 hours until they could be frozen in a -30C freezer before analysis. Visible symptoms of virus infection were recorded (presence/absence) and flower color classified as light purple (LP), dark purple (DP), white (W) or color breaking (CB) whenever petal tissue was present. Coordinates and qualitative information about habitat were noted as well.

### 2.2 Virus identification

During initial virus screening in 2016, we used enzyme linked immunosorbent assays (ELISA) to test whole-leaf samples for four virus species and one virus family: cauliflower mosaic virus (CaMV), turnip yellow mosaic virus (TYMV), cucumber mosaic virus (CMV), turnip mosaic virus (TuMV) and taxa in the family *Potyviridae*. In the June survey, we used both the general potyvirus assay (Agdia, Elkhart, IN) which tests for at least 73 potyviruses, as well as the specific TuMV triple antibody sandwich ELISA. By assaying for potyviruses generally, we maintained the largest pool of potential viral species to include in the virus discovery process. Subsequent detection assays in 2018 used only TAS-ELISA specific for TuMV because corresponding positive results between potyvirus and TuMV ELISA tests suggested that TuMV was the primary potyvirus present.

### 2.3 RNA extraction and deep sequencing for virus confirmation

In addition to whole-tissue assays for TuMV using TAS-ELISA, we collected leaf and petal tissue for deep sequencing to detect viruses without making *a priori* assumptions about expected virus taxonomy. In spring 2018, fresh tissue was collected from five individual hosts near Cornell University as i) three standardized leaf disks per plant and ii) the pigmented portion of approximately three to five unopened flower buds per plant. Leaf and petal tissue was homogenized separately using liquid nitrogen. In this first assessment of a full *H. matronalis* virome, four symptomatic individual plants that had both leaf mosaic and color breaking petals were included, and one asymptomatic individual was assayed as a control (N=5 individuals with two tissue samples sequenced per individual). Small and micro RNA was extracted from each tissue sample (Qiagen RNeasy Mini kit), quality checked using a fragment analyzer and DNase removed prior to library prep. Library prep and small RNA sequencing on an Illumina HiSeq was done at the Cornell University RNA Sequencing Core. Small RNA (sRNA) sequences (>22 nt) were selected and viral sequences were detected using the VirusDetect bioinformatic platform v1.6 (Zheng et al., 2017). The *Arabidopsis thaliana* genome was selected to subtract host-derived sRNA, and viral contigs assembled using Velvet in VirusDetect.

### 2.4 Common garden experiment

To partition the effects of genetic and environmental variation among host lines, we conducted a common garden experiment using 19 maternal genotypes of *H. matronalis* collected from across upstate New York. Seeds were collected from single plants in September 2016, vernalized during winter 2017 and planted in soil in March 2017. Ten individual seedlings from each of the 19 genotypes were included in the experiment, and all plants were grown under common greenhouse environmental conditions. Five of the ten individuals per genotype were exposed to wild type TuMV inoculum collected from a single infected, symptomatic *H. matronalis* plant in Tompkins County during summer 2016. Wild-type TuMV infection was confirmed using immunostrips and TAS-ELISA (Agdia, Elkhart, IN).Inoculum was frozen in a -30C freezer until the beginning of the experiment, then macerated with buffer and introduced to carborundum-abrased leaf tissue. The five remaining individuals per genotype were mock inoculated using distilled water and carborundum power. Inoculations and mock inoculations occurred approximately two weeks after seed germination. Two weeks after inoculation, a leaf sample was taken to test for TuMV infection, and the first week of phenotypic data was collected. For each of the next nine weeks, data were collected on the presence or absence of leaf symptoms (mottling, crinkling), the number of leaves present and the length of the longest leaf over time. Every other week, a new leaf was collected and stored for TAS-ELISA. Leaves collected from weeks two through five were assayed for TuMV infection and symptom development.

### 2.5 Host and virus distribution estimation

Presence data from across the realized host range was downloaded from the Global Biodiversity Information Facility (GBIF) in February 2021, cleaned and corrected so that each occurrence included in our study represented a host population rather than a host individual. To estimate the presence of TuMV in *H. matronalis*, we conducted an image analyses of all high-quality individual host occurrence records collected between 2013 and 2020. Presence data was included if the image clearly showed the presence of color breaking flower phenotypes.

### 2.6 Statistical methods

#### 2.6.1 Field surveys and TuMV prevalence

Field surveys were used to estimate the prevalence of TuMV in *H. matronalis* across upstate New York. In 2016, this was calculated by comparing the number of plants that tested positive for TuMV to the total number of plants sampled over the course of the survey. In 2018, we estimated TuMV prevalence at the host population scale by calculating the number of positive samples out of ten collected at each host population, compared to average prevalence across all samples. We compared positivity rates between regions using a chi-squared test with simulated p-values to create a distribution of expected results.

#### 2.6.2 Common garden experiment

We assessed the effect of host genotype on plant growth using generalized linear mixed models with a negative binomial distribution to test for interactive and additive differences between common garden host genotypes, populations, regions and treatment throughout and at the end of the experiment. Growth was estimated using mean values of total leaf area per plant by multiplying the number of leaves produced by the longest leaf length. GLMM models were implemented using the glmmTMB package (Brooks et al., 2017) and lme4 (Bates et al., 2015) in R software version 4.0.3 (R Core Team, 2020). We selected the best fitting model based on AIC values, in which fixed effects (survey week, treatment and genotype) and random effects (host identity) are compared across four evenly spaced sample weeks (weeks two, four, six and eight).

## 3 Results

**Fig. 2:**
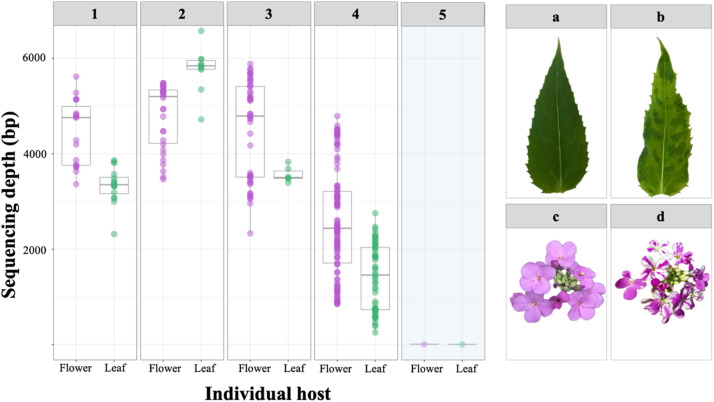
Symptomatic host individuals (panels 1, 2, 3 and 4 on the left) were infected with viral contigs that matched to TuMV; asymptomatic control (panel 5) did not yield any virus contigs. Symptomatic phenotypes were defined by leaf mottle (panel B) and color breaking petals (panel D), while asymptomatic individuals had solid pigmentation and regular leaf margins (panels A and C).

**Fig. 3:**
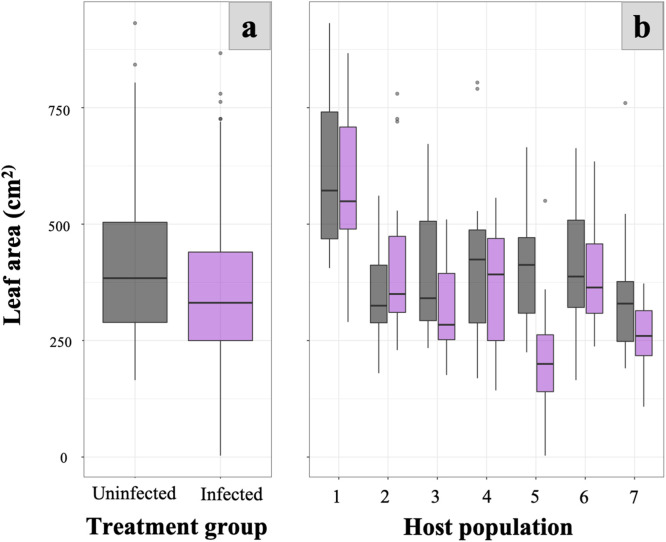
Leaf area estimate at the end of the common garden experiment. Host plant groups inoculated with TuMV are indicated in purple while control plants are in grey.

**Fig. 4:**
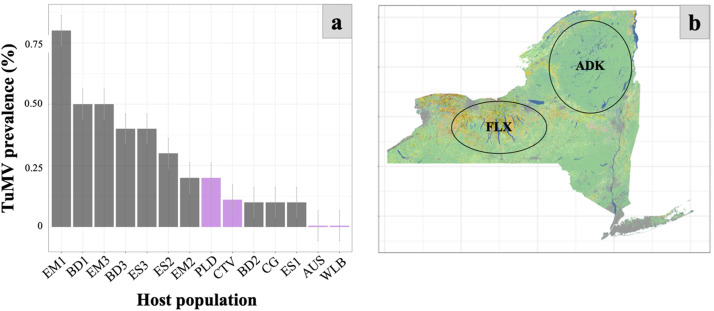
Field surveys of TuMV prevalence in *H. matronalis* in 2018 in upstate New York. Purple indicates results from the Adirondacks and grey represents results from the Finger Lakes. A. TuMV prevalence across sites. Bars represent the standard deviation around the mean (sd=0.06). B Landcover variation between the agricultural Finger Lakes and largely forested.

Our study provides evidence of widespread TuMV infection and tolerance in the wild host plant *H. matronalis* across host populations and landscape types. We show that the geographic extent of TuMV infection in the wild plant host *H. matronalis* is broad, and may be especially common in the Great Lakes region of North America (Fig. 1)*.* Furthermore, we confirm that the infection is systemic throughout host plants and is associated with asymmetrical color streaking in floral tissue (Fig. 2). Experimental infections suggest that there is considerable variability in host susceptibility to TuMV across populations across upstate New York, and especially within a population from Lake Placid. When environmental variation is eliminated, unequal performance across infected hosts grown under common conditions suggests that tolerance to TuMV may be genetically determined within wild *H. matronalis* lineages (Fig. 3). Finally, virus prevalence differs across host populations and between regions (Fig. 4). We suggest that the processes determining TuMV distribution and impact in wild hosts should be explored further.

### 3.1 Confirmation of TuMV infection in a widespread wild host

In two separate growing seasons, we tested 1,029 unique individual hosts for TuMV (786 in 2016, and 243 plants in 2018). Across years and samples, 17% of 921 *H. matronalis* hosts tested positive for TuMV. We found variable rates of infection in other sampled brassicas, including 4% (3 of 72) in *A. petiolata* and a single infection each in species *Sinapis arvenses* and *Brassica napus*.

Leaf mottling or crinkling was observed for 81.4% of all TuMV positive individuals of *H. matronalis* and 20.2% of TuMV negative samples. Furthermore, 13 of the 157 TuMV-positive *H. matronalis* individuals (8% of all positive tests) were marked as asymptomatic in the field, suggesting that infection does not always induce clearly visible symptoms. As such, it is clear that leaf symptoms alone are not consistent enough to provide definitive diagnostic power. We did not observe color breaking petals in plants that tested negative for TuMV, and thus consider virus-induced color breaking in petals to be a potential diagnostic tool for efficiently monitoring the presence of TuMV. However, the absence of leaf mottling or color breaking does not definitively indicate the absence of the virus.

In our 2018 field survey, we balanced host population size across upstate New York (Fig. 4). We found variation in the prevalence of TuMV by host population, but especially by region. Specifically, we found that TuMV prevalence depended on the region from which hosts were sampled, with far greater TuMV prevalence in the Finger Lakes region compared to the Adirondacks (χ^2^=8.06, p-value=.007). In the Finger Lakes region, the overall prevalence of TuMV in 2018 was 34.5% (39 infected of 113 sampled). In the Adirondack samples, the overall prevalence of TuMV in 2018 was 7.5% (3 infected of 40 sampled). Infection rates varied among populations from 0% to 11% in the Adirondacks and 10% to 39% in the Finger Lakes.

Deep sequencing of tissue collected in 2018 showed that no other viruses were detected in symptomatic tissue from *H. matronalis* individuals for which full viromes were built, and asymptomatic control tissue yielded no TuMV contigs (Fig. 2). While the small and localized sample size precludes generalization of these results across the *Hesperis*-TuMV pathosystem, it does indicate that the most common symptoms observed are not caused by coinfection, and that no other potyvirus was present. All of the symptomatic tissue, be it leaf or petal, had viral RNA most closely aligned with TuMV when compared to the National Center for Biotechnology Information (NCBI) virus genome database (Table S2).

### 3.2 Virus diversity in Hesperis matronalis in upstate New York

Of the 786 plants assayed in 2016, 88 were positive for TuMV, 6 plants in the late season survey were positive for CMV, and none were infected with CaMV or TYMV. All TuMV and CMV infections were detected in plants in the Finger Lakes region; no virus infections were detected in any plant hosts growing in the Adirondacks in 2016. A single plant tested positive for both CMV and TuMV in the late season survey, indicating that coinfections are not especially common.

Viromes built from deep sequencing of leaf and petal tissue from five individual hosts yielded only TuMV viral contigs. Tissue collections for sequencing were made from a single population of hosts, and the sample size is too small to be useful in predicting virus diversity across the broad range of *H. matronalis*.

### 3.3 Impacts of infection vary according to host provenance

When averaged across host genotypes, we observed a slight but significant decrease in leaf area for plants infected by TuMV compared to those uninfected by TuMV (p=0.016, Fig. 3A), confirming that infection by TuMV can impair growth in *H. matronalis*. To account for host factors that mediate disease severity, we also examined genotypic differences in leaf area under common growing conditions. In our common garden experiment, GLMM analysis indicated some genotypic effects on above-ground biomass (Fig. 3B). Since all plants were infected with the same wild type virus inoculum (thus holding virus diversity constant) and environmental conditions were equivalent across all plants, any variation in performance implicates host genotype in determining the severity of pathogenesis. We found that genotypes from Placid (Adirondack region) consistently performed significantly worse when infected by TuMV, whereas other genotypes did not perform differently (p-value=0.02, Fig. 3B). We conclude that i) the impact of infection is detectable at the host population scale, ii) most hosts are tolerant of TuMV infection, but iii) there is some genetic variation in host response to infection across host populations in upstate New York.

## 4 Discussion

Here we show that wild *H. matronalis* in the Finger Lakes region of New York is often infected by TuMV, that the infection typically results in visible symptoms useful for monitoring efforts, and that host genetics is relatively more important than host environment in determining the consequences of infection for host performance. We also find significant variation in *H. matronalis* tolerance to TuMV among host populations. Based on the current distribution and densities of *H. matronalis* across the United States (Fig. 1), our study may have implications for vegetable growers and oil crop producers in other regions who experience yield loss from TuMV. For example, mixed use landscapes akin to those found in the Finger Lakes occur across the Midwest and Great Lakes regions (Fig. S1; USDA CropScape, 2021), where high densities of *H. matronalis* populations co-occur with agricultural hosts (Fig. 1). Attempts to map and breed durable genetic resistance to TuMV infection in crops such as canola and Chinese cabbage have yielded mixed results (reviewed in (Li et al., 2019; Palukaitis and Kim, 2021; Walsh and Jenner, 2002), and the current ‘best bet’ for genetic control of TuMV is through introgression of the recessive, broad-spectrum resistance gene retr01 into cultivars (Nellist et al., 2022). There are not yet any commercial varieties available with durable resistance, however, so disease management at the field level is still critical for protecting crops. Given our results and the agroecological implications of high TuMV prevalence in *H. matronalis*, we suggest that farmers in the midwestern region of the United States may consider monitoring their hedgerows for visible signs of TuMV in *H. matronalis* as an early warning of TuMV prevalence, particularly in regions with high susceptible crop coverage. That said, our data do not provide justification for removal of *H. matronalis* or other potential wild hosts. Until TuMV spillover from wild *H. matronalis* populations into crop plants is measured directly, it may be more cautious to leave wild host populations intact rather than risk further disturbance and subsequent weed invasions, as was observed in an experimental removal study in Indiana (Pavlovic et al., 2009). Nonetheless, high prevalence of TuMV in populations of *H. matronalis* growing in agricultural regions warrants further research and monitoring to determine what, if any, role alternative hosts play in TuMV-associated yield losses.

Mixed-use landscape matrices increase the likelihood of spillover of both zoonotic pathogens (Plowright et al., 2021) and phytopathogens (Roossinck and García-Arenal, 2015), and percent crop cover in the Finger Lakes region, where TuMV prevalence is high in *H. matronalis*, is similar to that in the Great Lakes and upper Midwest regions of North America (Fig. S1). In these agroecological landscapes, common crops like canola grow near weedy species that may be reservoirs for viruses. The probability of spillover between hosts is especially notable for viruses with broad host ranges and non-persistent modes of transmission (Power and Mitchell, 2004), and there is evidence that landscape simplification generally increases the likelihood of viral disease emergence as a result of spillover between wild and crop hosts (Roossinck and García-Arenal, 2015). So, just as the possibility of crop infection via spillover increases, we also show that TuMV-*Hesperis* co-occurrence is common in agricultural landscapes (Fig. 1). Our results suggest that even relatively diverse agroecological environments, such as the Finger Lakes region, may be more favorable for virus spread and persistence than largely forested ecosystems.

While serological assays and RNA sequencing results provide compelling evidence that there is a link between TuMV infection and color breaking petals, we acknowledge the possibility of alternative explanations. Spontaneous mutations in plant genomes, particularly those caused by insertions of transposable elements (McClintock, 1953), can disrupt anthocyanin pigmentation in petals resulting in a color breaking phenotype similar to the one we report here (Iida et al., 2004; Slotkin and Martienssen, 2007). Such mutations are heritable and have been useful in horticultural breeding of unusual cultivars in many flowering plants (Morita and Hoshino, 2018; Nishijima et al., 2022; Yamagishi et al., 2018). However, infection with some viruses can also exogenously manipulate the expression of genes such that pigment biosynthesis is disrupted. While we acknowledge that transposable elements or other host mutations may induce phenotypes similar to those we attribute to TuMV infection, we did not find hosts that displayed color breaking but were uninfected by TuMV. Furthermore, *Potyviridae* is a diverse family of plant viruses that infect many host species and induce various phenotypic traits, but one of the more common characteristics associated with potyvirus infections is color breaking petals (Valverde et al., 2012). Furthermore, we successfully induced the color-breaking phenotype in an experimental host plant grown from purple-morph seeds (Fig. S2). Therefore, we conclude that the most parsimonious explanation for the disrupted pigmentation in TuMV-positive individuals is that the virus is impacting anthocyanin deposition. Further research into the mechanism underlying this pattern is necessary, and we suggest that the phenological timing of infection may be an important factor determining which infected plants display color breaking, and to what degree. Finally, if we are correct that color breaking petal phenotypes are the result of viruses, this phenotype is not heritable because TuMV is not commonly vertically transmitted, if at all (Cobos et al., 2019; Montes et al., 2019). Examination of specific potential fitness costs, such as reduced pollinator visits or seed germination, are necessary before drawing conclusions regarding selective pressures shaping the evolutionary ecology of *H. matronalis-*TuMV interactions.

Ecological consequences of non-native plant species are mixed, but ruderal species like *H. matronalis* are often assumed to be associated with loss of native biodiversity (Powell et al., 2011; Vilà et al., 2011). However, there are few and inconclusive studies regarding the competitive impact of *H. matronalis* on native plants despite its classification as ‘invasive’ in thirteen state lists (EDDMaps, Brown County Native Woodlands Project; Pavlovic et al., 2009; National Park Service). While *H. matronalis* can outcompete certain native perennials in controlled environments (Hwang and Lauenroth, 2008), a field-based study of *H. matronalis* removal showed no impact on native plant cover, but did lead to encroachment by non-native woody plants (Pavlovic et al., 2009). Thus blanket generalizations regarding the invasiveness of *H. matronalis* in the introduced geographical range do not accurately represent plant interactions across environments, and more research is required before implementing removal programs. Furthermore, the presence of *H. matronalis* may benefit other organisms, such as bees, syrphid flies and some lepidopterans that gain pollen from the abundant floral resources produced by *H. matronalis in* early spring (Majetic et al., 2009; Mitchell and Ankeny, 2001).

It is possible that host range expansions and invasions facilitate the distribution of associated viruses in wild plant communities (Litchman, 2010; Mitchell et al., 2006; Rúa et al., 2011). Indeed, characterization of recombination rates and virus genomic diversity suggests that the spread of TuMV across Europe and Asia was facilitated through a wild orchid (Nguyen et al., 2013), with some spatial proximity to historical trade routes as humans distributed plant hosts (Kawakubo et al., 2021). Analogous research documenting TuMV ecology, evolution and distribution is absent from North America, though TuMV infections cause significant damage to crops (Li et al., 2019; Tomlinson, 1987). Given the non-persistent mode of TuMV transmission by a variety of widely-distributed aphid species and the breadth of potential host species common across global plant communities, it is reasonable to predict that the realized distribution of TuMV across the Great Lakes region, particularly in human-disturbed environments, is greater than previously documented. As such, spillover between wild and agricultural hosts is likely. To better examine the consequences of TuMV across habitats, it is critical to first understand which widespread wild hosts are viable, and the mechanisms that determine infection outcomes across environments.

We found that the severity of disease varies by both host environment and genotype with most host genotypes displaying tolerance to the virus, but one host lineage suffering significantly lower total estimated leaf area and eventual mortality when infected. The most susceptible genotypes were collected from the host population where no infections were found in the field (Placid population in the Adirondack Mountains), which implicates population-level genetic variation in determining the outcome of virus infection. It is possible that hosts that typically experience low levels of virus infection in the field are relatively more susceptible when experimentally infected, as has been observed in other studies (Anderson et al., 2004; Fiallo-Olivé and Navas-Castillo, 2019; Jones, 2009). In this case, it would follow that populations with consistently higher TuMV prevalence may have experienced selection over generations, leading to resistance or tolerance to infection. We did not find resistance, but we found that most host lineages across upstate New York displayed tolerance to TuMV. This result indicates that the likelihood of TuMV-induced disease emergence in wild plants varies and should be studied at the scale of host populations.

The *H. matronalis* -TuMV pathosystem is widespread across upstate New York and the Great Lakes region, and portions of the midwestern agricultural region of North America have similar landscape characteristics. We recommend further research to determine whether these common hosts are a source or recipient of TuMV spillover between crops and wild hosts, and whether or not management of wild *H. matronalis* hosts can reduce TuMV-induced disease in crops. Collaboration with farmers and extension agents would facilitate a better assessment of the risk facing farmers in areas where *H. matronalis* is especially abundant.

## 5 Conclusions

Here, we demonstrate that the *Hesperis*-TuMV pathosystem is common and widespread, particularly in the Great Lakes region of North America, and that it presents a tractable system in which to study plant-virus interactions across environments. In addition, we confirm Ford's hypothesis that color breaking in petals is correlated with virus infection, which can be a complementary method for detecting the presence of TuMV in unmanaged hosts, in addition to molecular diagnostics. Surveys of smaller *H. matronalis* populations from the Adirondacks region indicate both lower host density and apparent absence of the virus, though this should be monitored. Further work exploring the spatial extent of this pathosystem, mechanisms driving virus dispersal between host populations, and possible virus strain diversity are needed to understand what, if any, role this widespread weedy host might play in crop loss from TuMV.

## Funding

This research was funded by a grant to E.M.L. by the Kieckhefer family through Cornell University.

## Authors contribution

E.M.L. conceptualized this study, conducted field research, analyzed results and wrote the first draft of the manuscript. J.P. was instrumental in developing the field system and conducting lab and greenhouse work. L.J. collected greenhouse data and provided manuscript edits. A.G.P. supervised, supported and edited all components of this research.

## Declaration of Competing Interest

All authors declare no competing interests.

## Data Availability

Data will be made available on request.

## Appendix Supplementary materials

Image, application 1

Image, application 2
