# Supplementary material for: Wild and weedy Hesperis matronalis hosts turnip mosaic virus across heterogeneous landscapes in upstate New York
Source: Virus Res. 2022 Nov 28;323:199011. doi: 10.1016/j.virusres.2022.199011 (PMC10194180; doi:10.1016/j.virusres.2022.199011)
Supplement: Supplementary file 1 [file mmc1.docx]

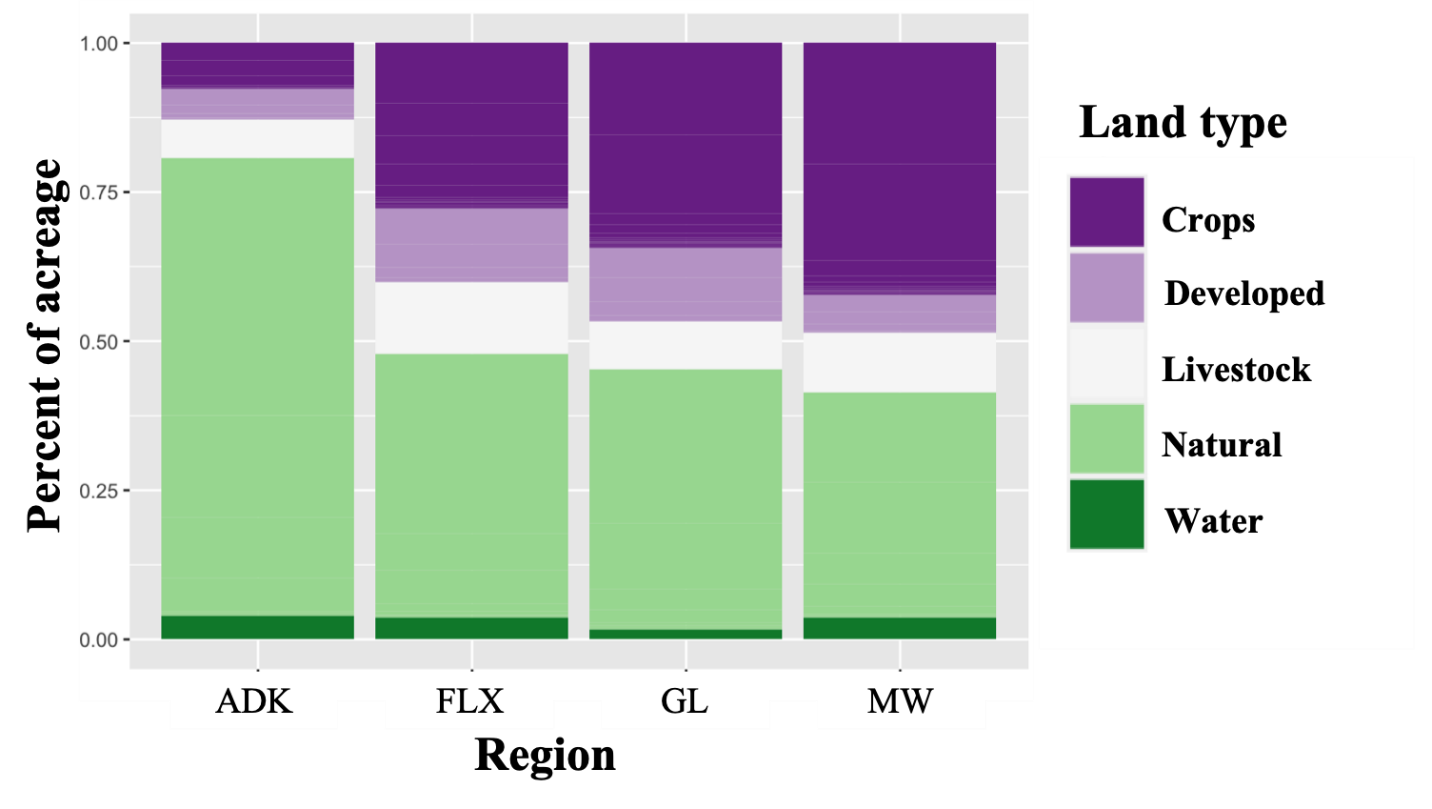


Figure S1: Comparison of landscape characteristics in four bioregional areas in which *H. matronalis* is a common weed. The most common landcover type is bolded for each of the four regions. Categorization of USDA CropScape data from 2021 by Lombardi.


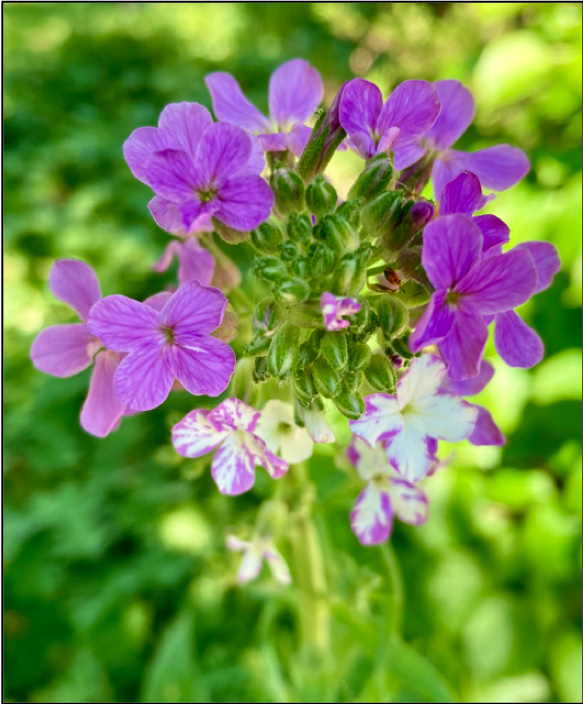


Figure S2: Photograph of experimentally-induced color breaking phenotype in purple morph *H. matronalis* infected with wild type TuMV.

**Supplementary Experiment S2**

*Symptom induction experiment*

To test for causation between TuMV infection and visible symptom development, we infected *H. matronalis* plants grown from commercially-sourced violet morph seed (Eden Brothers, Arden, NC) (N=20). Half of the experimental plants were mock inoculated using distilled water, and half were infected with wild type TuMV collected from a wild host population in upstate New York. Plants were grown to the rosette stage, then exposed to simulated winter conditions (low light and temperature in a dry cooler) to prompt flowering. Unfortunately, this experiment was disrupted during spring 2020 due to the SARS-CoV-2 pandemic and all plants were moved from campus growing facilities to an outdoor area. We used exclosures to minimize the chance of uncontrolled infection from insect vectors, and watered regularly, but mortality from the rapid change in growing environment led to very high mortality. Of the few surviving plants, only three flowered. One of the three was in the infection group, and two were in the control group. Neither control plants produced color breaking symptoms, and the one infected plant did produce color breaking symptoms (Figure S2). The results of this experiment cannot be reported without acknowledging significant sources of unmeasured variability. However, we consider this preliminary evidence that there is a causative relationship between TuMV infection and color breaking symptoms in *H. matronalis* flowers.


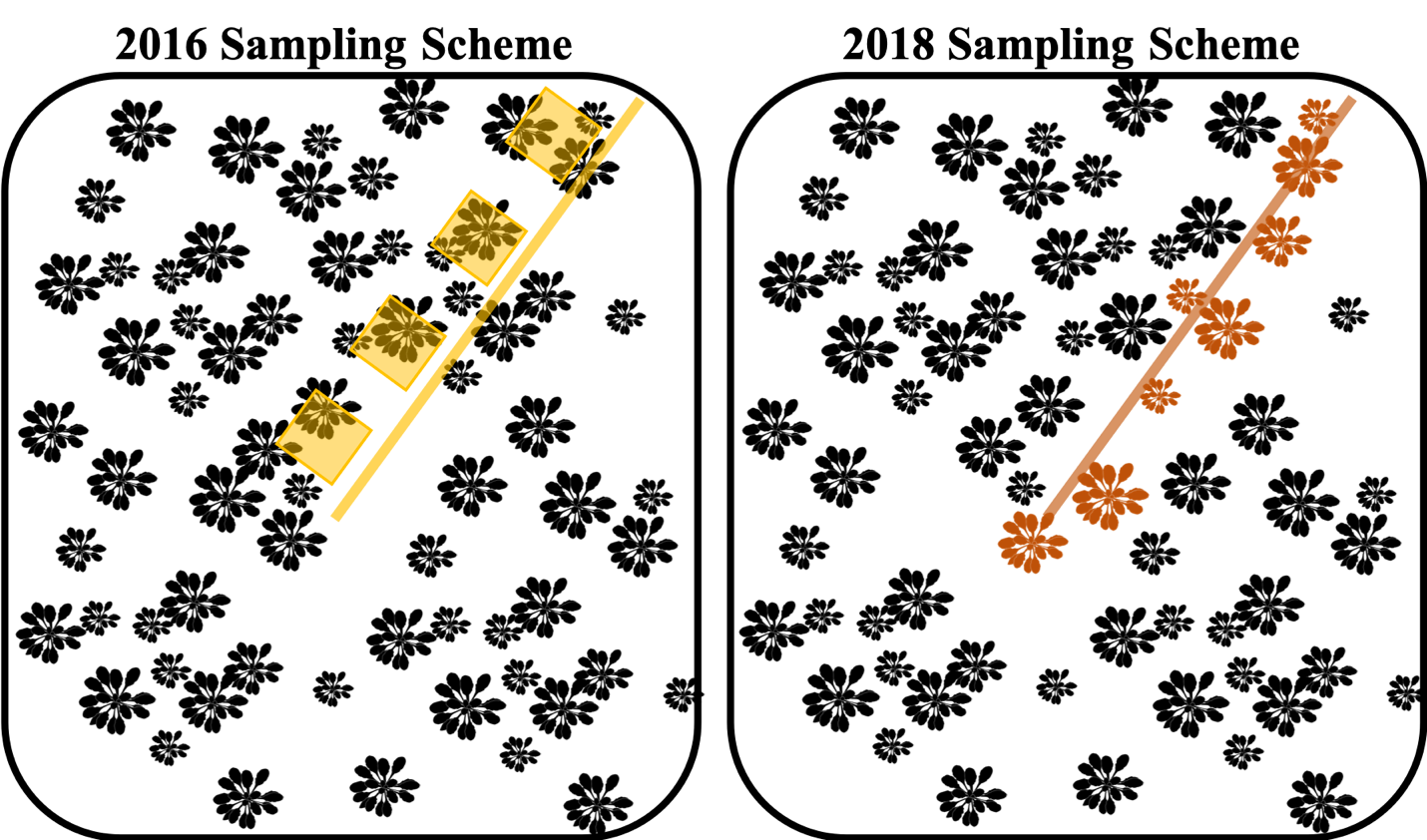


**Figure S3:** Diagrammatic depiction of field sampling methods for years 2016 versus 2018. Transects from the middle to edge of host populations were used each year. In 2016 we sampled all hosts within 1 meter x 1 meter quadrats laid along the transect (N=number of plants that fell randomly within quadrats). In 2018 we sampled ten random hosts touching the transect (N=10 hosts per population). Note that Adirondack host populations were of insufficient size to replicate the exact method in 2018 and instead we collected ten individuals from center to edge however we could.

| **Code** | **Population** | **Region** | **Year** | **Latitude** | **Longitude** | **No. samples** | **No. positive** | **Positivity rate** |
| --- | --- | --- | --- | --- | --- | --- | --- | --- |
| Aus | Ausable | ADK | 2018 | 44.55952625 | -73.44822597 | 11 | 0 | 0.005 |
| CV | Clintonville | ADK | 2018 | 44.4942488 | -73.5278975 | 9 | 1 | 0.111 |
| Placid | Placid | ADK | 2018 | 44.2785743 | -73.9768336 | 10 | 2 | 0.2 |
| Wills | Willsboro | ADK | 2018 | 44.3660028 | -73.3921944 | 10 | 0 | 0.005 |
| BDA | Black Diamond A | FLX | 2018 | 42.4580778 | -76.5205444 | 10 | 5 | 0.5 |
| BDB | Black Diamond B | FLX | 2018 | 42.4580778 | -76.5205444 | 10 | 1 | 0.1 |
| BDC | Black Diamond C | FLX | 2018 | 42.4580778 | -76.5205444 | 10 | 4 | 0.4 |
| CG | Cornell Gardens | FLX | 2018 | 42.4620029 | -76.4446492 | 10 | 1 | 0.1 |
| ESA | East Shore A | FLX | 2018 | 42.4728869 | -76.5041392 | 10 | 1 | 0.1 |
| ESB | East Shore B | FLX | 2018 | 42.4728869 | -76.5041392 | 10 | 3 | 0.3 |
| ESC | East Shore C | FLX | 2018 | 42.4728869 | -76.5041392 | 10 | 4 | 0.4 |
| EM | Etna Mill | FLX | 2018 | 42.4771600 | -76.38685182 | 10 | 8 | 0.8 |
| ENP | Etna Preserve | FLX | 2018 | 42.4771600 | -76.38685182 | 10 | 2 | 0.2 |
| Etna | Etna/highway 13 | FLX | 2018 | 42.4771600 | -76.38685182 | 10 | 5 | 0.5 |

Table S1: Population information from structured TuMV surveys of wild *H. matronalis* populations in 2018.

Table S2: Results from RNA-Seq and VirusDetect analyses. Accession numbers are provided for NCBI SRA database queries, along with sequencing results and the TuMV variants to which isolates most closely matched. Plants 1 through 4 were visibly symptomatic with both leaf and petal symptoms. Plant 5 was visibly asymptomatic.
